# Comparison of amphotericin B deoxycholate in combination with either flucytosine or fluconazole, and voriconazole plus flucytosine for the treatment of HIV-associated cryptococcal meningitis: a prospective multicenter study in China

**DOI:** 10.1186/s12879-022-07665-z

**Published:** 2022-08-08

**Authors:** Ting Zhao, Xiaolei Xu, Yushan Wu, Wei Zhang, Qin Zeng, Yanqiu Lu, Tongtong Yang, Guoqiang Zhou, Jianhua Yu, Ke Lan, Vijay Harypursat, Yaokai Chen

**Affiliations:** 1 Clinical Research Center, Chongqing Public Health Medical Center, Chongqing, China; 2 Division of Infectious Diseases, Chongqing Public Health Medical Center, 109 Baoyu Road, Shapingba, Chongqing, 400036 China; 3 Division of Infectious Diseases, Public Health Clinical Center of Chengdu, Sichuan, China; 4 Division of Infectious Diseases, The First Hospital of Changsha, Hunan, China; 5 Division of Infectious Diseases, Xixi Hospital of Hangzhou, Zhejiang, China; 6 Department of Infectious Disease, Longtan Hospital of Guangxi Zhuang Autonomous Region, Guangxi, China

**Keywords:** Amphotericin B deoxycholate, Cryptococcal meningitis, HIV, Voriconazole, Fluconazole, Flucytosine

## Abstract

**Background:**

The most appropriate alternative to induction therapy for HIV-associated cryptococcal meningitis (CM) remains unclear when standard treatment is unavailable, inaccessible, intolerable, or ineffective.

**Methods:**

A prospective, multi-centre cohort study was conducted to analyze the data of 156 HIV-infected patients with CM who were treated with amphotericin B deoxycholate (AmB-D) + flucytosine (5FC), voriconazole (VCZ) + 5FC, or AmB-D + Fluconazole (Flu) as induction regimens. Clinical efficacy, cumulative mortality, and adverse effects were compared among the three treatment groups.

**Results:**

Fewer deaths occurred by week 4 and week 10 among patients receiving AmB-D + 5FC than among those receiving AmB-D + Flu [4 (5.1%) vs. 8 (16.0%) deaths by week 4; hazard ratio, 1.8; 95% confidence interval [CI], 1.0 to 3.3; *p* = 0.039; and 8 (10.3%) vs. 14 (28.0%) deaths by week 10; hazard ratio, 1.8; 95% CI, 1.1 to 2.7; *p* = 0.008, respectively]. AmB-D plus 5FC was found to result in significantly higher rates of cerebrospinal fluid (CSF) culture sterility (57.6% vs. 34% by week 2; 87.9% vs. 70% by week 10; *p* < 0.05 for both comparisons). However, the differences in CSF culture sterility and mortality between the VCZ + 5FC group and the AmB-D + 5FC group were not statistically significant. VCZ plus 5FC had a significantly advantageous effect on the incidence of new AIDS-defining illness and length of hospital stay, compared with AmB-D plus 5FC. Laboratory adverse events (grade 3 or 4), such as severe anemia, were less frequent with VCZ + 5FC use than with AmB-D combined with 5FC or Flu use.

**Conclusion:**

Our results suggest that AmB-D combined with 5FC remains the more efficacious induction regimen compared to AmB-D plus Flu, and that VCZ + 5FC might be a potential alternative when the standard regimen is not readily available, accessible, tolerated, or effective.

*Clinical Trials*: Registration number, ChiCTR1900021195. Registered 1 February 2019, http://www.chictr.org.cn/showproj.aspx?proj=35362.

## Introduction

Cryptococcal meningitis (CM) remains an important contributor to HIV-associated deaths, accounting for an estimated 15% of overall HIV mortality globally [1]. Treatment of CM comprises three therapeutic stages: the induction, the consolidation, and the maintenance phases [2]. For induction therapy for CM, amphotericin B deoxycholate (AmB-D) administered intravenously, combined with oral flucytosine (5FC), is the current internationally recommended treatment regimen [3, 4]. The objective of induction treatment is to rapidly and dramatically decrease the mycotic load in cerebrospinal fluid (CSF), and is thus critical for patient survival [5].

Challenges remain to the implementation of this treatment recommendation in resource-limited settings. For example, 5FC is neither easily accessible nor generally affordable in areas with high HIV-related CM burden [6, 7]. In addition, standard doses of AmB-D are not well tolerated due to the potential for substantial nephrotoxicity [8]. In contrast, Flu is now more readily available in a generic form and via donation programs, and is associated with low rates of adverse events [9, 10]. An elevated dose of Flu is therefore advocated as an alternative to 5FC for combination therapy with AmB-D; however, the efficacy of the combination of high dose Flu combined with AmB-D remains debatable [11].

Voriconazole (VCZ) is a new generation triazole with good CSF penetration and a wide-spectrum of activity against pathogens [12–14]. However, not many published research studies have, as yet, explored VCZ as a potentially useful therapeutic drug for the induction or maintenance phases of treatment for CM [15, 16]. Most data with respect to its use are derived from refractory case reports, with about 50% treatment success observed [12, 17, 18]. Currently, the role of VCZ for the management of CM has not been definitively established.

Thus, the present study was designed to compare two currently recommended regimens and a novel treatment strategy, with the aim of discovering an appropriate alternative to the preferred regimen for primary induction therapy. The options we chose for comparison included AmB-D plus 5FC, VCZ plus 5FC, and AmB-D plus Flu.

## Methods

### Study design and participants

We conducted a prospective multicenter study of HIV-related CM in six Chinese centers: Chongqing Public Health Medical Center, Public Health Clinical Center of Chengdu, The First Hospital of Changsha, The First Affiliated Hospital of Zhejiang University, Guangxi Longtan Hospital, and Xixi Hospital of Hangzhou. HIV-infected patients were eligible for enrollment if they were diagnosed with CM, either confirmed by CSF India ink stain, cryptococcal culture in blood or CSF, or cryptococcal antigen (CrAg) testing in CSF; were 18 years of age or older; and were willing to be contacted for study surveillance. Patients were excluded if their alanine aminotransferase (ALT) levels were > 5 times the upper limit of normal, hemoglobin levels were < 60 g/L (reference range: 120–160 g/L for males, 110–150 g/L for females), creatinine levels were > 1.5 times the upper limit of normal, were pregnant, were lactating, or developed any past adverse effects to any of the specific drugs used in our trial. All patients in the cohort were enrolled between January 2019 and December 2020.

### Study procedures

At enrollment, participants completed interviews which gathered information regarding their baseline characteristics (including socio-demographic information, clinical symptoms, past medical history), and basic medical tests (such as CSF profiles, blood tests, and imaging examinations, etc.). Lumbar punctures (LPs) for CSF examination were typically performed on days 0, 7, and 14 after admission. Additionally, patients with elevated intracranial pressure (ICP) were treated with therapeutic LPs until the opening pressure decreased to below 20 cmH_2_O.

Each eligible participant was treated with one of the following three induction therapy regimens: Regimen 1 consisted of AmB-D (0.5–0.7 mg per kilogram per day administered intravenously) plus 5FC (100 mg per kilogram per day) for approximately 4 weeks; Regimen 2 consisted of VCZ (200 mg twice daily; 400 mg twice on day 1) plus 5FC (100 mg per kilogram of body weight per day) given orally for approximately 4 weeks; Regimen 3 consisted of AmB-D (0.5–0.7 mg per kilogram per day administered intravenously) plus Flu (800 mg per day) for approximately 4 weeks. Induction treatment regimens were chosen by the attending physician in accordance with current Chinese clinical guidelines.

All participants were treated and observed in-hospital for a minimum of 4 weeks during induction therapy. The results of laboratory blood tests were monitored regularly during this period. Patients in all treatment arms were subsequently switched to oral Flu [800 mg per day for 8 weeks (consolidation phase), followed by 200 mg per day (maintenance phase)]. Patients treated with rifampicin were not assigned to Group 2 due to the potential for harmful drug interaction between rifampicin and VCZ.

Local Chinese prescribing guidelines for HIV were used to prescribe antiretroviral regimens. Participants who were already taking antiretroviral therapy (ART) when they were diagnosed with CM, continued with their usual treatment. For ART-naïve participants or those patients taking an ineffective ART regimen, the recommendation was to initiate or change the ART regimen four to six weeks after induction antimycotic treatment [19].

Study visits occurred at weeks 1, 2, 3, 4, 10, 22, and 46 after commencement of CM induction. Assessment of safety was done via participant interviews which questioned participants regarding possible emergence of new clinical symptoms, and clinical examination of participants for assessment of emergence of possible new clinical signs, and included laboratory tests and recording of clinical adverse events. Compliance to prescribed treatment was assessed by reference to clinical patient diaries that were accurately completed by medical staff.

### Outcomes and definitions

The primary outcomes for the comparison of the three study treatment strategies were all-cause mortality at 10 weeks and CSF culture sterility at 2 weeks. Secondary outcomes included all-cause mortality at weeks 2, 4, and 46, CSF culture sterility at 10 weeks, culture-positive relapse, re-hospitalization related to induction therapy, 24-week and 46-week clinical response, therapeutic success at week 10, new AIDS-defining illness, emergence of paradoxical immune reconstitution inflammatory syndrome (IRIS), and cumulative hospitalized days over 46 weeks. We compared the incidence of new neurological events, seizures, rashes, hyperpyrexia, respiratory system disorders, gastrointestinal disorders, and any adverse laboratory event (grade 3 or 4) in the two study groups. CM relapse was defined as a re-positive CSF *Cryptococcus* culture result obtained after induction therapy. The definition of IRIS has been previously described in published literature [20]. The Division of AIDS Table for Grading the Severity of Adult and Pediatric Adverse Events: December 2017, was used to assess and record adverse events which occurred during the course of this study.

### Statistical analysis

Baseline participant characteristics and outcomes in groups were compared via the Pearson Chi squared (χ^2^) test or Fisher’s exact test (for categorical variables), and the one-way analysis of variance (ANOVA) or the Kruskal–Wallis test (for continuous variables). All-cause mortality at two, four, and ten weeks was compared between the groups using log-rank tests. Kaplan–Meier plots were also drawn, and Cox regression models with treatment as a predictor were used to derive hazard ratios and two-sided 95% confidence intervals (CL). Other outcomes were compared across treatment groups with the Chi-squared, Kruskal–Wallis, Fisher’s exact, or Mann–Whitney U test, as appropriate. All statistical analyses were performed using Stata software, Version 16 (StataCorp. 2019. Stata Statistical Software: Release 16. College Station, Texas, USA: StataCorp LLC), GraphPad Software (GraphPad Prism, La Jolla, California, United States), Version 6.0, and Statistical Package for the Social Sciences (IBM-SPSS Software for Windows) software, Version 24 (IBM Corp., Armonk, New York, USA).

## Results

### Baseline characteristics

**Fig. 1:**
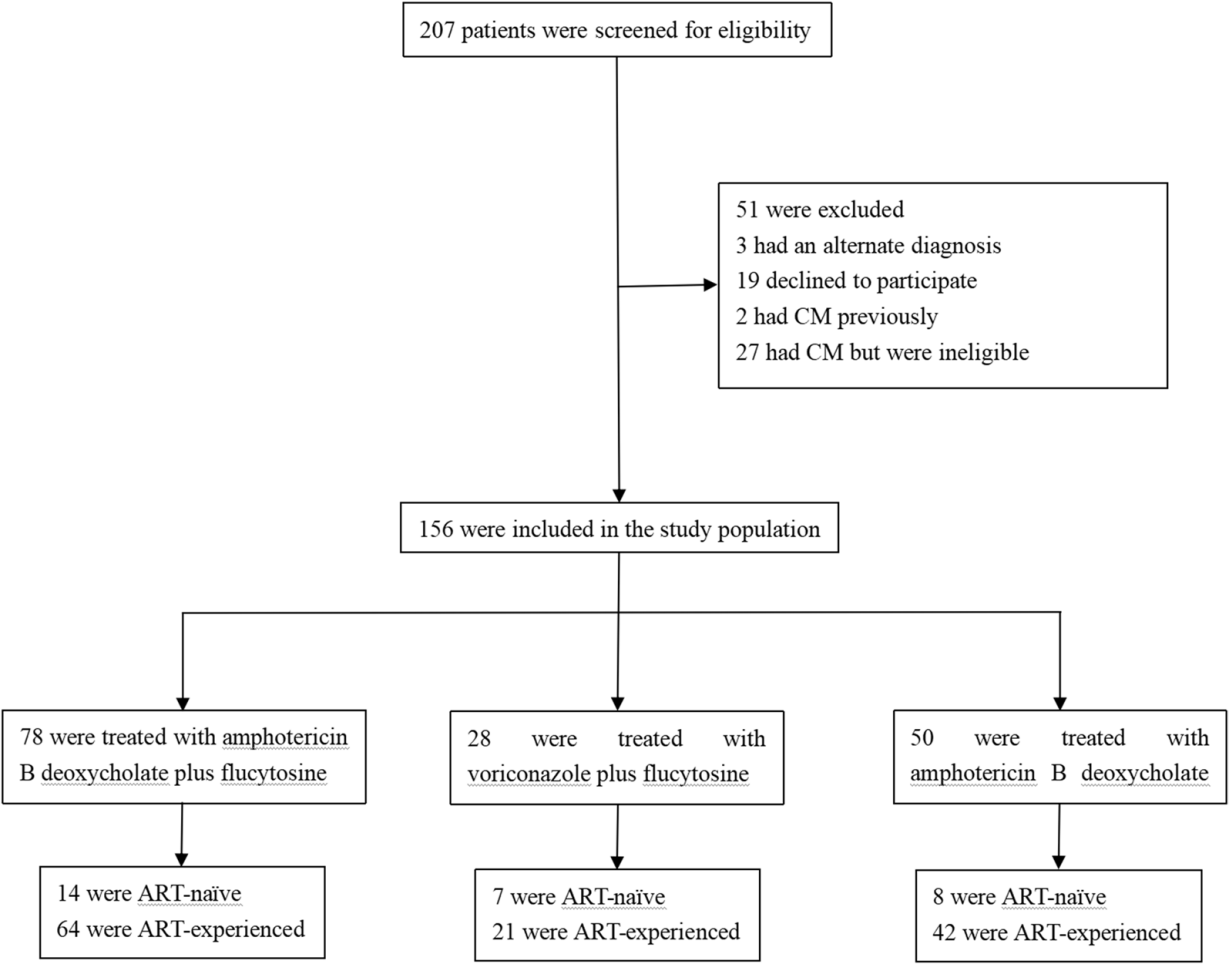
Flowchart of patient selection. ART denotes antiretroviral therapy; CM denotes cryptococcal meningitis

During the study period, 207 patients with HIV-associated CM were eligible for study participation, and 156 (75%) were fully enrolled (i.e., met screening criteria for our study and completed follow-up to the end of the trial) and were included in the analysis population for treatment comparison (Fig. 1). Of these 156 eligible participants, 78 were assigned to group 1 (the AmB-D + 5FC group), 28 to group 2 (the VCZ + 5FC group), and 50 to group 3 (the AmB-D + Flu group).

Baseline characteristics of patients were similar between the three groups, and showed the severity of HIV-associated immunocompromise in the entire population (Table 1). CSF cultures yielded *Cryptococcus neoformans* in 143 of 156 patients (91.7%). In total, 81.4% (127/156) of participants confirmed that they were, or had previously been, exposed to antiretroviral drugs. Participants who had not previously ever taken ART initiated antiretrovirals at a median initiation point of 35 days (range, 27 to 39 days) after enrollment.

**Table 1:** Baseline characteristics of study participants

	AmB-D + 5-FC (N = 78)	VCZ + 5FC (N = 28)	AmB-D + Flu (N = 50)	*p-*value^*^
*Demographics*				
Male—no. (%)	62 (79.5)	20 (71.4)	36 (72)	0.534
Median age (IQR)—years	42 (34, 52.3)	41 (34, 51)	45 (34.8, 57)	0.568
*Clinical characteristics*				
BMI, mean ± SD	20.3 ± 2.7	19.5 ± 2.5	20.4 ± 2.9	0.419
Median WBC (IQR)—(10^9^/L)	4.9 (3.5, 6.7)	4.4 (3, 6.9)	3.8 (3.2, 5.4)	0.176
Hemoglobin (g/L), mean ± SD	111.6 ± 20.8	105.8 ± 23.8	109 ± 18.1	0.426
Median creatinine level (IQR)—µmol/L	57.1 (47.7, 66.2)	66.0 (51.2, 87)	59.9 (49.3, 72.2)	0.111
Median CD4 + T-cell count (IQR)—cells/µL	28 (12, 53)	24 (12.5, 59)	36 (13, 44.3)	0.981
Current ART use—no. (%)	14 (17.9)	7 (25)	8 (16)	0.606
Receiving TB therapy—no. (%)	7 (9)	2 (7.1)	7 (14)	0.601†
Symptoms—no. (%)				
Headache	57 (73.1)	21 (75)	32 (64)	0.464
Fever	55 (70.5)	19 (67.9)	31 (62)	0.604
Nausea	40 (51.3)	12 (42.9)	18 (36)	0.231
Vomiting	43 (55.1)	13 (46.4)	17 (34)	0.065
Visual field impairment	5 (6.4)	4 (14.3)	5 (10)	0.388^†^
Impaired consciousness	13 (16.7)	5 (17.9)	6 (12)	0.730^†^
Opportunistic infection—no. (%)				
PCP	6 (7.7)	1 (3.6)	1 (2)	0.443^†^
CMV	4 (5.1)	4(14.3)	7 (14)	0.152^†^
*CSF profile*				
Median ICP (IQR)—cm H_2_O	25 (16, 33)	25.5 (14, 34.3)	26.5 (14.5, 40)	0.856
CSF ICP > 25 cm H_2_O—no. (%)	37 (47.4)	14 (50)	28 (56)	0.639
Median CSF WBC (IQR)—10^6^/L	23.5 (6, 103)	3 (0, 58)	17.5 (9.5, 44.5)	0.106
Median CSF glucose level (IQR)—mmol/L	2.4 (1.6, 195.7)	2.6 (1.9, 3.7)	2.2 (1.2, 3.3)	0.266

*BMI* body mass index, *WBC* white blood cells, *ART* antiretroviral therapy, *TB* tuberculosis, *CSF* cerebrospinal fluid, *PCP* pneumocystis pneumonia, *CMV* cytomegalovirus infection, *ICP* elevated intracranial pressure, *IQR* interquartile range, *AmB-D* amphotericin B deoxycholate, *5FC* flucytosine, *VCZ* voriconazole, *Flu* fluconazole

^*^p-values from the Pearson χ^2^ or Fisher’s exact test for categorical variables and the one-way ANOVA or Kruskal–Wallis test for continuous variables, unless otherwise specified. ^†^Fisher exact test was used

### All-cause mortality

**Fig. 2:**
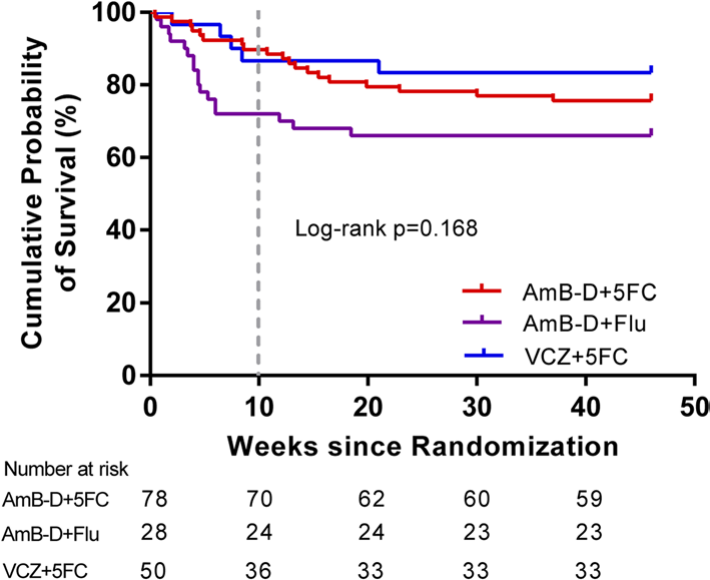
Kaplan–Meier survival estimates for the three treatment groups over 46 weeks

Kaplan–Meier curves for survival up to 46 weeks for the entire study population according to their treatment strategy are shown in Fig. 2. By 10 weeks, eight patients (10.3%) treated with AmB-D + 5FC had succumbed, in comparison to 14 patients (28.0%) using AmB-D + Flu, and four patients (14.3%) treated with VCZ + 5FC (Table 2). AmB-D + 5FC treatment has been linked to significant reductions in the risk of death by week 10 in the primary outcome (hazard ratio, 1.8; 95% CI, 1.1 to 2.7; *p* = 0.008); this benefit was initially seen at 4 weeks (*p* = 0.039). A lower number of participants being treated with VCZ + 5FC died, when comparison was made with deaths in participants treated with AmB-D + Flu; however, this difference in mortality was calculated to not be statistically significant (hazard ratio, 2.2; 95% CI,0.7 to 6.8; *p* = 0.142). The 2-week mortality was 2.6% (2/78) for patients receiving AmB-D + 5FC, 8.0% (4/50) for patients receiving AmB-D + Flu, and 3.6% (1/28) for those receiving VCZ + 5FC; differences in mortality rates at week 2 between groups were calculated to not be mathematically significant. The hazard ratios for death at 2 weeks, as compared with the AmB-D + 5FC group were 1.4 (95% CI, 0.1 to 15.2) in the VCZ + 5FC group and 1.8 (95% CI, 0.8 to 4.2) in the AmB-D + Flu group; the corresponding hazard ratios for death at 4 weeks were 0.7 (95% CI, 0.1 to 6.3) and 1.8 (95% CI, 1.0 to 3.3), respectively.

**Table 2:** All-cause mortality and CSF culture sterility in the three treatment groups

	No. of patients % (95% CI), by regimen			Hazard Ratio, % (95% CI)^†^					
Outcomes	Group 1, AmB-D + 5FC (N = 78)	Group 2, VCZ + 5FC (N = 28)	Group 3, AmB-D + Flu (N = 50)	Group 2 *vs* Group 1	*p-* value	Group 3 *vs.* Group 1	*p-* value	Group 2 *vs.* Group 3	*p-* value
Mortality at 10 week No. of deaths % (95% CI)	8 10.3 (3.5 to 17.0)	4 14.3 (1.3 to 27.2)	14 28.0 (15.6 to 40.4)	1.4 (0.4 to 4.6)	0.582	1.8 (1.1 to 2.7)	0.008	2.2 (0.7 to 6.8)	0.142
Mortality at 2 weeks No. of deaths % (95% CI)	2 2.6 (− 0.9 to 6.1)	1 3.6 (− 3.3 to 10.4)	4 8.0 (0.5 to 15.5)	1.4 (0.1 to 15.2)	0.791	1.8 (0.8 to 4.2)	0.156	2.3 (0.3 to 20.9)	0.435
Mortality at 4 weeks No. of deaths % (95% CI)	4 5.1 (0.2 to 10)	1 3.6 (− 3.3 to 10.4)	8 16.0 (5.8 to 26.2)	0.7 (0.1 to 6.3)	0.747	1.8 (1.0 to 3.3)	0.039	4.7 (0.6 to 37.9)	0.105
				Treatment difference, % (95% CI)^‡^					
CSF culture negative at 2 weeks No. of patients % (95% CI)	38 57.6 (45.7 to 69.5)	13 48.1 (29.3 to 67.0)	17 34.0 (20.9 to 47.1)	− 7.6 (− 31.9 to 12.9)	0.407	− 23.6 (− 41.3 to − 5.8)	0.012	14.1 (− 8.8 to 37.1)	0.224
CSF culture negative at 10 weeks No. of patients % (95% CI)	58 87.9 (80.0 to 95.8)	21 77.8 (62.1 to 93.5)	35 70 (57.3 to 82.7)	− 10.1 (− 27.6 to 7.4)	0.221^*^	− 17.8 (− 32.8 to − 2.9)	0.017	7.8 (12.4 to 30.0)	0.465

*CI* confidence interval, *p*-values for the between-group differences in all-cause mortality were calculated using a log-rank test

*Fisher’s exact test was used to compare rate of CSF culture sterility between groups

^†^Hazard ratios are shown for all outcomes except for the estimated treatment difference in CSF culture sterility rate

^‡^Differences between mortality rates are given as percentage points. The upper limit of the two-sided 95% confidence interval is equivalent to that of the one-sided 97.5% confidence interval

### CSF culture sterility

Thirty-eight (57.6%) of 66 patients in the AmB-D + 5FC arm achieved sterile CSF at 2 weeks, compared to 13 (50%) of 27 in the VCZ + 5FC arm and 17 (34%) of 50 in the AmB-D + Flu arm; sterile CSF rates achieved at 2 weeks between the AmB-D + 5FC arm and AmB-D + Flu arm were found to be statistically significantly different (*p* = 0.012). The treatment difference for mycological outcome (negative CSF culture) at 2 weeks, as compared against the AmB-D + 5FC arm, were -7.6% (95% CI, -31.9 to 12.9) for the VCZ + 5FC arm and 14.1% (95% CI, -8.8 to 37.1) for the AmB-D + Flu arm. Consequently, VCZ + 5FC was found to be as effective as, but was not superior to, AmB-D + 5FC with respect to mycological outcome at week two. At 10 weeks, CSF cultures were negative in 87.9% of patients receiving AmB-D + 5FC, 77.8% of patients receiving VCZ + 5FC, and 70% of those receiving AmB-D + Flu (Table 2). None of the observed differences among the three groups were calculated to be mathematically significant (treatment difference for group 2 vs. group 1, − 10.1% (95% CI, − 27.6 to 7.4%), and treatment difference for group 3 vs. group 1, − 17.8% (95% CI, − 32.8 to − 2.9%).

### Outcomes in the three treatment groups

The survival benefit at 46 weeks seen for patients receiving AmB-D + 5FC as compared with those receiving AmB-D + Flu, was not marked (*p* = 0.237, Table 3). Treatment with VCZ + 5FC failed to bestow a survival advantage in comparison to treatment with AmB-D + Flu (*p* = 0.129). Clinical responses at 2 weeks, defined as improvement of fever, headache, and meningeal signs, occurred in 42.6% of patients receiving AmB-D + 5FC, in comparison to 53.8% of patients receiving VCZ + 5FC, and 40.9% of patients who received AmB-D + Flu. Fifty-two (89.7%) of 58 patients in group 1, 18 (81.8%) of 22 patients in group 2, and 26 (74.3%) of 35 patients in group 3 had clinical responses at week 10 (Table 3).

**Table 3:** Outcomes related to prognosis in the three treatment groups

	No. (%) of patients, by regimen			*p*-value		
Event	Group 1, AmB-D + 5FC (N = 78)	Group 2, VCZ + 5FC (N = 28)	Group 3, AmB-D + Flu (N = 50)	Group 2 *vs* Group 1	Group 3 *vs* Group 1	Group 2 *vs* Group 3
Mortality at 46 weeks	19 (24.4)	5 (17.9)	17 (34)	0.481	0.237	0.129
Clinical responses: week 2	29 (42.6)	14 (53.8)	18 (40.9)	0.330	0.856	0.294
Clinical responses: week 10	52 (89.7)	18 (81.8)	26 (74.3)	0.450	0.051	0.509
Therapeutic success: week 10	62 (91.2)	20 (76.9)	33 (75)	0.085^*^	0.020	0.856
Culture-positive relapse	4 (5.1)	1 (3.6)	1 (2)	1.0^*^	1.0^*^	1.0^*^
Re-hospitalization	20 (25.6)	5 (17.9)	13 (26)	0.405	0.964	0.413
New AIDS-defining illness	13 (16.7)	0 (0)	8 (16)	0.019^*^	0.921	0.045^*^
Paradoxical IRIS	7 (9)	1 (3.6)	2 (4)	0.678^*^	0.481^*^	1.0^*^
Cumulative hospitalized days over 46 weeks	46 (27.5,67)	28.5 (19.8, 46.3)	39.5 (27, 51)	0.040	0.174	0.158

*IRIS* immune reconstitution inflammatory syndrome. CM relapse was defined as a re-positive CSF *cryptococcus* culture after induction therapy

^*^Fisher’s exact test was used

Therapeutic success was defined as a combined mycologic and clinical response at 10 weeks. Success was achieved in 91.2% of patients who took AmB-D + 5FC, in 76.9% of those who took VCZ + 5FC, and in 75% of those who took AmB-D + Flu. Neither clinical response nor therapeutic success differed in a statistically significant manner among the three treatment groups. The incidence of culture-positive relapse, re-hospitalization, and paradoxical IRIS was similar among all three treatment groups. IRIS occurred infrequently, with seven cases observed in the AmB-D + 5FC group (Group 1), one case observed in the VCZ + 5FC group (Group 2) and two cases observed in the AmB-D + Flu group (Group 3). Patients receiving VCZ + 5FC (Group 2) had a significantly lower chance of developing a new AIDS-defining illness, as compared with those receiving AmB-D in combination with either 5FC or Flu (*p* = 0.019 and *p* = 0.045 for comparison of groups 1 and 3 with group 2, respectively). Of note, VCZ combined with 5FC significantly reduced the cumulative length of hospital stay at 46 weeks, when compared with the AmB-D + 5FC group.

### Adverse effects of treatments

All regimens produced frequent adverse clinical events, and this was a likely to be a consequence of the severely immunocompromised status prevalent in the studied patient cohort (Table 4). New neurological events included impaired consciousness, abnormal mental status, hallucinations, visual disturbances, etc. The occurrence of these events was not calculated to be significantly different between the three groups (*p* = 0.322). The number of patients having seizures or skin rashes was similar in all treatment groups. Gastrointestinal disorders were uncommon, and incident rates were similar in the three groups. There were 5 cases of hyperpyrexia in the AmB-D + 5FC group, 1 case in the VCZ + 5FC group, and 4 cases in the AmB-D + Flu group. Five and six patients in the AmB-D + 5FC and AmB-D + Flu groups experienced respiratory system disorders, respectively.

**Table 4:** Adverse events

Event	No. (%) of patients, by regimen			*p-value*
	Group1, AmB-D + 5-FC (N = 78)	Group 2, VCZ + 5FC (N = 28)	Group 3, AmB-D + Flu (N = 50)	
Any clinical adverse event—no. of patients (%)	31 (39.7)	12 (42.9)	24 (48)	0.654
New neurologic sign or symptom—no. of patients (%)	19 (24.4)	11 (39.3)	14 (28)	0.322
Seizure—no. of patients (%)	3 (3.8)	2 (7.1)	5 (10)	0.413^*^
Rash—no. of patients (%)	7 (9)	1 (3.6)	5 (10)	0.687^*^
Hyperpyrexia—no. of patients (%)	5 (6.4)	1 (3.6)	4 (8)	0.830^*^
Respiratory system disorder	5 (6.4)	0 (0)	6 (12)	0.127^*^
Gastrointestinal disorder	2 (2.6)	1 (3.6)	2 (4)	0.852^*^
Any adverse laboratory event—no. of patients (%)				
Grade 3 or 4	49 (62.8)	7 (25)	34 (68)	< 0.001
Grade 3	38 (48.7)	5 (17.9)	27 (54)	0.005
Grade 4	25 (32.1)	2 (7.1)	15 (30)	0.033
Anemia—no. of patients (%)				
Grade 3	19 (24.4)	2 (7.1)	9 (18)	0.135
Grade 4	16 (20.5)	0 (0)	13 (26)	0.012
Hypokalemia—no. of patients (%)				
Grade 3	12 (15.4)	1 (3.6)	8 (16)	0.239^*^
Grade 4	2 (2.6)	0 (0)	0 (0)	0.686^*^
Leukopenia—no. of patients (%)				
Grade 3	5 (6.4)	0 (0)	5 (10)	0.213^*^
Grade 4	3 (3.8)	0 (0)	1 (2)	0.820^*^
Hyperuricemia—no. of patients (%)				
Grade 3	5 (6.4)	0 (0)	4 (8)	0.355^*^
Grade 4	4 (5.1)	1 (3.6)	1 (2)	0.856^*^
Thrombocytopenia—no. of patients (%)				
Grade 3	5 (6.4)	2 (7.1)	4 (8)	0.918^*^
Grade 4	2 (2.6)	1 (3.6)	2 (4)	0.852^*^
Increase Creatinine—no. of patients (%)				
Grade 3	2 (2.6)	0	4 (8)	0.263^*^
Grade 4	0	0	0	–
Other grade 3 or 4 adverse event—no. of patients (%)^‡^	7 (9)	1 (3.6)	5 (10)	0.687^*^

All clinical adverse events and all laboratory events of grades 3 or 4 were classified as severe adverse events

*p-values correspond to overall comparisons among the three groups using Fisher’s exact test

^‡^Other adverse events occurred in less than 3% of patients, except for hyponatremia (which occurred in 3.2% of all patients)

Laboratory-defined adverse events (grade 3 or 4) were less frequent in the VCZ + 5FC group than in either the AmB-D + 5FC group or the AmB-D + Flu group (*p* < 0.001 for the overall comparison). The *p*-values for the occurrence of grade 3 and grade 4 adverse events in the three treatment groups were 0.005 and 0.033, respectively. The most frequently occurring adverse events observed in the three groups were anemia, hypokalemia, thrombocytopenia, leukopenia, and hyperuricemia. Grade 4 anemia occurred more commonly among patients taking AmB-D with either 5FC or Flu, compared to those receiving VCZ + 5FC (20.5% and 26%, respectively, vs. 0%; *p* = 0.012 for the overall comparison). A grade 4 hypokalemia developed in only two patients, and this complication was most likely circumvented as a result of preemptive electrolyte replacement that was administered to all patients receiving AmB-D. There were fifteen cases of grade 3 or 4 hyperuricemia recorded, and elevated uric acid levels occurred in 11.5% of the patients who were taking the AmB-D + 5FC regimen, in 3.6% of those taking VCZ + 5FC, and in 10% of those taking the AmB-D + Flu regimen. Grade 4 leukopenia was observed in 3.8% of patients taking AmB-D + 5FC, in 0% of those taking VCZ + 5FC, and in 2% of those taking AmB-D + Flu regimen. A grade 3 or 4 thrombocytopenia developed in 9% of patients in the AmB-D + 5FC group, in 10.7% of patients in the VCZ + 5FC group, and in 12% of patients in the AmB-D + Flu group. A grade 3 increase in creatinine level developed in 2 of 78 participants (2.6%) in the AmB-D + 5FC group and in 4 of 50 participants (8%) in the AmB-D + Flu group. There were no grade 3 or 4 increases in creatinine levels in the VCZ + 5FC group.

## Discussion

In this trail, we attempted to compare the efficacy and safety of AmB-D plus 5FC, VCZ plus 5FC, and AmB-D combined with high-dose Flu for management of acute CM in HIV-infected patients. The results of this multisite study indicate that there are no statistically significant differences between combination therapy with VCZ plus 5FC and the current standard treatment of AmB-D + 5FC with respect to primary clinical outcomes, and that 5FC in combination with AmB-D is associated with lower mortality (week 4, 10) and higher culture conversion rates (week 2, 10) than that of Flu in combination with AmB-D among those patients with data available.

In a randomized, comparative clinical trial evaluating either VCZ, Flu, and 5FC in combination with AmB-D as induction therapy for HIV-associated CM, the early fungicidal activity (EFA) of VCZ in combination with AmB-D was not significantly different from the EFA of high-dose Flu or 5-FC combined with AmB-D [15]. Yao et al., observed that the rate of response to VCZ treatment was significantly higher than the response rates to a combination of AmB-D [or liposomal amphotericin B (L-AmB)] and Flu, and a combination of AmB (or L-AmB) and 5FC (100% vs 67.9% and 27.3%, respectively; *p* < 0.01) [16]. Even so, clinical data regarding the efficacy of VCZ for the treatment of CM remains relatively limited, and large-scale, comparative experimental and clinical investigations are warranted to support a definitive recommendation with respect to its use in the context of CM.

In vitro and animal model data supports a role for VCZ in the management of cryptococcosis via results indicating that broad-spectrum triazoles have excellent efficacy against *Cryptococcus neoformans* [21]. In animal model studies of CM, it was observed that VCZ did reduce fungal burden, and prolonged the survival time of infected mice, with good CSF penetration [22–24]. Several case studies have suppressed outcomes of CM using VCZ, and VCZ has been suggested as an alternative therapeutic choice for patients whose *Cryptococcus* isolates develop secondary fluconazole resistance [18, 25].

Interestingly, it appears that benefits of VCZ in combination with 5FC were observed in data for both new AIDS-defining illnesses and length of hospital stay, and an absence of a significant increase in the occurrence of serious adverse effects (either clinical or laboratory) was observed to be associated with this therapeutic strategy. There appeared to be no grade 3 or 4 increases in ALT levels that could have been related to VCZ, which is in keeping with findings from previous clinical trials [15]. In addition, VCZ appeared to be safe and well tolerated at an oral dose of 200 mg taken twice per day for approximately 4 weeks. However, utilization of VCZ for the management of CM is limited by frequent drug-drug interactions, especially with commonly prescribed ART, as well as cost and availability.

In comparison with the therapeutic regimen of AmB-D plus Flu (Flu is a reliable and universally available second-line antifungal agent), AmB-D in combination with 5FC was associated with decreased mortality in our study; Moreover, the mathematical difference between these groups did achieve statistical significance. The survival benefit with the use of AmB-D plus 5FC that we observed in the present study concurs with the findings of one other double-blind multicenter study [26]. Additionally, AmB-D combined with 5FC resulted in significantly higher rates of CSF sterilization at two weeks than with use of AmB-D combined with Flu (57.6% vs. 34%; treatment difference for two groups, − 23.6% [95% CI, − 25.0 to − 22.2]), which is consistent with the findings of one randomized, open-label, three-group trial of induction regimens to treat CM, overseen by Day et al. [10]. Contrarily, one previous study found no significant differences in rates of *Cryptococcus* clearance from patient CSF between AmB-D plus 5FC and AmB-D plus Flu [15]. Furthermore, AmB-D plus Flu (800 mg/day) has been selected as the standard antifungal induction drug regimen in one multicenter investigation being conducted on the timing of ART initiation subsequent to initiation of induction therapy for CM in Africa [27].

The better-performing regimens in our study included 5FC. It appears that this more efficacious partner drug is especially useful when shorter courses of AmB-D are used. Improving the opportunity to use 5FC can potentially produce material reductions in the number of deaths attributable to CM. 5FC may also possess other qualities, such as a long-lasting “postantibiotic effect” compared to Flu, in addition to swift fungicidal activity [11]. Another likely reason for the discernable variance between the two groups is that Flu has been shown to antagonize the anti-*Cryptococcus* properties of AmB-D, as observed in one past in vitro investigation [28].

It should be noted that in this study, the dose of AmB-D used was 0.5–0.7 mg/kg/d for induction therapy (i.e., the dose recommended in current Chinese guidelines), which is lower than the recommended dose of 0.7–1.0 mg/kg/d used in current international guidelines [3, 4]. Although some studies suggest that high doses of AmB-D could improve patient outcomes [29], our study showed a mortality rate of 17.2% at 10 weeks, significantly lower than that of CM patients in Uganda (approximately 46%) [20], African CM patients in general (35%) [11], and Taiwanese CM patients (32%) [30]. In addition, the recommended dosage of AmB-D for the treatment of CM remains controversial, and several past investigations have reported that a lower prescribed dose of AmB [including that of liposomal (L)-AmB] results in a favorable safety profile with comparable benefit for the prevention or treatment of fungal infections [31–34].

We acknowledge several limitations to our study, which include the failure to enroll the originally calculated sample size and the non-randomized regimen allocation procedure, which may have led to a degree of bias. Therefore, some degree of circumspection should be exercised when generalizing our results to the entire Chinese population. Further large multicenter randomized controlled studies are warranted to extract accurate data with respect to induction regimens for the treatment of CM in the Chinese population. In addition, VCZ concentrations in plasma were not quantified and monitored during our study, nor was susceptibility testing routinely undertaken for follow-up cultures. Consequently, the likelihood of development of drug resistance to Flu during drug therapy, thus inducing treatment failure, cannot be confidently ruled out. Also, serial quantitative cultures of *Cryptococcus* in CSF should ideally be performed to accurately assess and compare the EFA of different treatment regimens, and could guide the setting of priorities with regards to which new drugs or drug combinations should proceed to much larger clinical investigations [35, 36]. Furthermore, we allowed the inclusion of those patients who were taking or who had taken ART in the past, and this may have had a confounding effect on the efficacy of fungal therapy.

In summary, the combination therapy of AmB-D plus 5FC was the most effective option as induction therapy for Chinese HIV-infected CM patients in our trial. Our results also suggest that the orally administered antifungal duo of VCZ and 5FC could be considered as an alternative option for the induction phase of CM treatment in the absence of availability of AmB-D or in circumstances where AmB-D cannot be administered safely.

## Data Availability

The datasets supporting the conclusions of this article are included within the article.
